# Unilateral recurrent macular hole in a patient with retinitis pigmentosa: a case report

**DOI:** 10.1186/1752-1947-7-69

**Published:** 2013-03-14

**Authors:** Miriam García-Fernández, Joaquín Castro-Navarro, Antonio Bajo-Fuente

**Affiliations:** 1 Department of Ophthalmology, Central University Hospital of Asturias, C/DionisioRidruejo, nº5, 11ºD, CP: 33007, Oviedo, Asturias, Spain; 2 Ophthalmologic Clinic Bajo and Castro, Vitreoretinal Center, Oviedo, Asturias, Spain

## Abstract

**Introduction:**

Several macular complications related to abnormalities of the vitreoretinal interface have been classically attributed to retinitis pigmentosa of which cystoid macular edema is the most common. Other less frequent complications are as follows: epiretinal membranes, vitreomacular traction syndrome and macular holes.

**Case presentation:**

A 64-year-old woman, with the previous diagnosis of retinitis pigmentosa, was referred to our department with a complaint of central visual loss in her left eye for 12 months. A fundoscopy and optical coherence tomography examination revealed the presence of a macular hole more than 500 microns in diameter. The patient underwent 20-gauge pars plana vitrectomy. Closure of the hole was observed after surgery, but reopening occurred at 2 years postoperatively.

**Conclusion:**

The pathogenesis of macular hole formation in patients with retinitis pigmentosa is unclear. Surgical outcomes may not always be favorable, and the possibility of reopening must be taken into account, even after a long time.

## Introduction

Several macular complications related to abnormalities of the vitreoretinal interface have been classically attributed to retinitis pigmentosa (RP) of which cystoid macular edema (CME) is the most common. Other less frequent complications are as follows: epiretinal membranes (ERM), vitreomacular traction (VMT) syndrome and macular holes (MHs) [1-4].

The detection of these abnormalities is crucial because they can significantly reduce the visual acuity in patients with RP whose central vision is usually well preserved until the late stages of the disease. The exact prevalence of these alterations is unknown, but we can say that the presence of a MH in patients affected by RP varies from 0.4% [5] to 10% [1]. The possibility of spontaneous resolution of a full-thickness MH in a patient with RP was once described in the literature [6] but, generally, a therapeutic approach based on vitrectomy is necessary [1,7].

We report a case of MH closure after pars plana vitrectomy (PPV) and late reopening, in a patient with RP.

## Case presentation

A 64-year-old woman was referred to our department with a complaint of central visual loss for 12 months in her left eye (LE). She had been diagnosed with RP at the age of 28 years.

**Figure 1:**
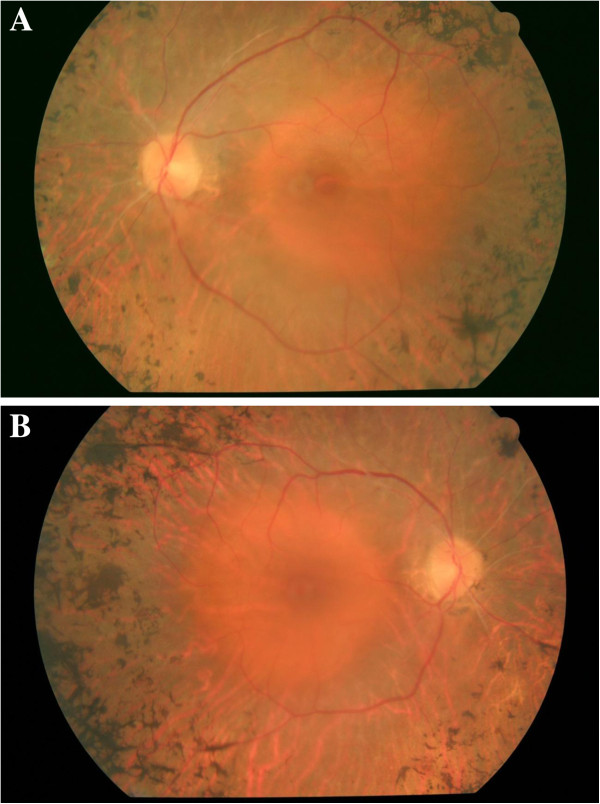
**Fundus photograph of left eye at baseline examination, showing the peripheral pigmentary changes related to the retinitis pigmentosa, and the macular round appearance corresponding to a macular hole with a yellow ring corresponding to the presence of subretinal fluid. **Visual acuity: 0.1 (decimal notation) **(A)**. Fundus photograph of right eye showing similar peripheral pigmentary changes to left eye, but with a normal macular appearance. Visual acuity: 0.8 (decimal notation) **(B)**.

At first examination, her best corrected visual acuity (BCVA) was 0.1 (decimal notation) in her LE, and 0.8 in her right eye (RE). An examination of the anterior segment was unremarkable. A fundoscopy examination of her LE revealed the presence of a stage-4 MH with a cuff of subretinal fluid surrounding it. A posterior vitreous detachment was observed. Narrowing of the retinal arterioles, waxy yellow appearance of the disk and hyperpigmentation in a bone-spicule configuration in the midperipheral retina were also observed in both eyes. However, the macular appearance of her RE was normal (Figures 1A and 1B).

An electroretinogram showed reduced scotopic and photopic responses in both eyes. Perimetry revealed the presence of an annular scotoma in both eyes, associated to central scotoma in her LE.

**Figure 2:**
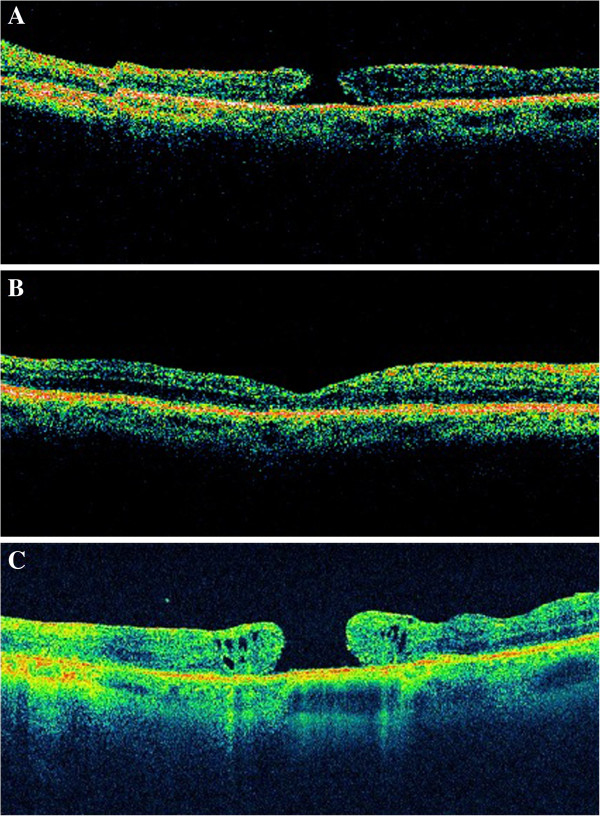
Optical coherence tomography prior to vitrectomy (A), 2 months later (showing the closure of the macular hole) (B) and 2 years after surgery (C), demonstrating the presence of a full-thickness macular hole.

Time-domain optical coherence tomography (Stratus OCT™, Carl Zeiss) confirmed the presence of a full-thickness MH in her LE (Figure 2A).

**Figure 3:**
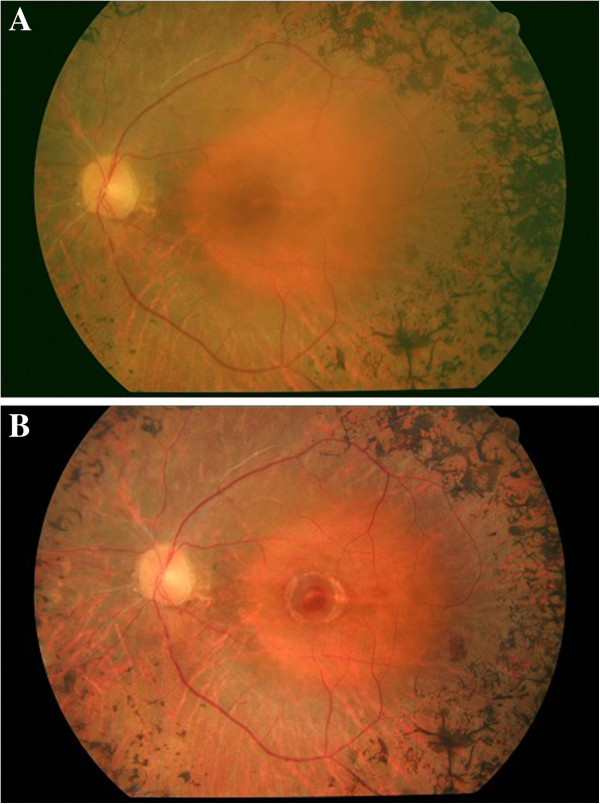
**Fundus photograph of left eye 2 months after vitrectomy, showing the closure of the macular hole. **Visual acuity: 0.4 (decimal notation) **(A)**. Fundus photograph 2 years after pars plana vitrectomy. A break in the central retina corresponding to the macular hole and a yellowish ring surrounding the hole due to the subretinal fluid can be observed. Visual acuity: 0.1 (decimal notation) **(B)**.

Therefore, we decided to perform 20-gauge PPV. Peeling of the internal limiting membrane (assisted by the dye Brilliant Blue G, Brilliant Peel®) in the area around the macular hole (2 disc diameters), and 25% sulfur hexafluoride intraocular gas fill were performed. Face down positioning for 5 days was also advised. At 2 months after the vitrectomy, her BCVA was 0.4 (decimal notation). Fundoscopy (Figure 3A) and OCT (Figure 2B) confirmed the hole closure, but severe macular atrophy was observed.

After 6 months, her BCVA was 0.1. A biomicroscopy showed the presence of a posterior subcapsular cataract. Fundoscopy and OCT confirmed the closure of the MH. Therefore, crystalline extraction with phacoemulsification (Infiniti Alcon Systems) was performed, and no complications occurred. However, BCVA after cataract surgery did not improve.

The patient came to our center 2 years after the vitrectomy complaining of mild central visual acuity loss in her LE. Her BCVA at this time was 0.1 in the LE and 0.8 in the RE. A fundoscopy (Figure 3B) and OCT examination (Figure 2C) demonstrated the reopening of the previously closed full-thickness MH and the presence of a ring of subretinal fluid and intense retinal atrophy surrounding the hole.

Because of the guarded prognosis, and the high likelihood that a reopening had occurred again due to the severe retinal atrophy around the hole, we decided not to perform surgery.

## Discussion

Before the advent of OCT, CME and other macular pathologies such as MHs were underdiagnosed [1]. CME among patients with RP was reported to be 10% to 20% [8]. With the introduction of the OCT, the prevalence of CME in patients with RP was described to be between 26% [1] and 38% [9].

The presence of MH in patients affected by RP varies from 0.4% [5] to 10% [1], with less than 10 papers about it in the literature.

Although the pathogenesis of macular edema in these patients has not been elucidated, it has been postulated that CME is due to a dysfunction of the retinal pigment epithelium (RPE) leading to a failure of the pumping mechanism and leakage of fluid through the RPE. However, the presence of a VMT might also contribute to CME formation due to mechanical traction of the posterior vitreous, as it can also occur in healthy patients.

Furthermore, patients with RP have a degenerative vitreous with collapse of vitreous gel and posterior vitreous detachment, which may facilitate all these vitreomacular interface changes [1].

Vitreoretinal surgery has no influence over the RPE dysfunction and, despite this, good outcomes after PPV have been reported. This fact makes us think that the mechanism of development of CME can differ between patients with RP, with VMT being responsible for CME formation in some patients, and inflammation and dysfunction of RPE pumping in others.

Something similar might occur with MH formation in these patients. The rupture and posterior fusion of the cysts from a CME can obviously contribute to the development of a MH. However, are there any other mechanisms implicated in the formation of a full-thickness MH in patients with RP?

The possibility of spontaneous resolution of a full-thickness MH in a patient with RP was once described in the literature [6] but, generally, a therapeutic approach based on vitrectomy is necessary.

There are few papers in the peer-reviewed literature about MH surgery in patients with RP. Hagiwara *et al*. [1] detected the presence of MH in three out of 622 eyes with RP, and performed surgery on two of them because one of the patients declined surgery. One of the patients obtained MH closure and vision improvement by single PPV and in the other patient repeated PPVs were necessary for MH closure, with reduced visual acuity after surgery related to RPE atrophy.

Jin *et al*. [7] reported the results after vitreoretinal surgery for management of four patients with MHs (one associated with a retinal detachment) and RP. Significant improvement of visual acuity was recorded for the three patients, with no change in visual acuity in the patient with retinal detachment.

Reopening of MHs after a primary successful surgery has been attributed to the contractile effect of postoperative ERM [10]. However, this does not seem to be the case in our patient as there was no evidence of ERM in OCT.

To the best of our knowledge, this is the first case which reports a closure and later reopening of a full-thickness MH in a patient with RP. Was the retinal atrophy surrounding the hole the main reason for its reopening, and are there unknown inflammatory causative factors in these patients which facilitate the recurrence?

Should we highlight in the preoperative counseling the guarded prognosis in cases in which vitrectomy is planned for patients with RP for MH repair?

## Conclusion

The pathogenesis of MH formation in patients with RP is unclear. Surgical outcomes are not always favorable, and this is why vitreous surgery may be carefully considered, and the possibility of reopening of the MH must be taken into account, even after a long time. However, it may be difficult to know whether there is a higher tendency for reopening in patients with RP and MH compared to those without RP. Further studies are warranted to confirm or refute these findings.

## Consent

Oral and written informed consent was obtained from the patient for publication of this case report and any accompanying images. A copy of the written consent is available for review by the Editor-in-Chief of this journal.

## Competing interests

The authors declare that they have no competing interests.

## Authors’ contributions

JCN and ABF photographed and interpreted the pathologic findings, and performed all the surgeries. MGF drafted the article, and analyzed and interpreted the patient data. All authors have made substantive intellectual contributions to this study and to the manuscript and have read and approved the final manuscript.
